# The Role of Parental Capacity for Medical Decision-Making in Medical Ethics and the Care of Psychiatrically Ill Youth: Case Report

**DOI:** 10.3389/fpsyt.2020.559263

**Published:** 2020-10-23

**Authors:** Ewa D. Bieber, Gail A. Edelsohn, Maria E. McGee, Julia Shekunov, Magdalena Romanowicz, Jennifer L. Vande Voort, Alastair J. S. McKean

**Affiliations:** ^1^Department of Psychiatry and Psychology, Mayo Clinic, Rochester, MN, United States; ^2^Community Care Behavioral Health Organization, UPMC Insurance Division, Pittsburgh, PA, United States; ^3^Department of Psychiatry, Creighton University School of Medicine, Omaha, NE, United States

**Keywords:** medical neglect, child and adolescent psychiatry, decisional capacity, harm principle, ethical dilemma

## Abstract

**Introduction:** Parents/legal guardians are medical decision-makers for their minor children. Lack of parental capacity to appreciate the implications of the diagnosis and consequences of refusing recommended treatment may impede pediatric patients from receiving adequate medical care. Child and adolescent psychiatrists (CAPs) need to appreciate the ethical considerations relevant to overriding parental medical decision-making when faced with concerns for medical neglect.

**Methods:** Two de-identified cases illustrate the challenges inherent in clinical and ethical decision-making reflected in concerns for parental capacity for medical decision-making. Key ethical principles are reviewed.

**Case 1:** Treatment of an adolescent with an eating disorder ethically complex due to the legal guardian's inability to adhere with treatment recommendations leading to the patient's recurrent abrupt weight loss.

**Case 2:** Questions of parental decisional capacity amid treatment of an adolescent with schizoaffective disorder raised due to parental mistrust of diagnosis, disagreement with treatment recommendations, and lack of appreciation of the medical severity of the situation with repeated discharges against medical advice and medication nonadherence.

**Discussion:** Decisions to question parental capacity for medical decision-making when risk of imminent harm is low but concern for medical neglect exists are controversial. Systematic review of cases concerning for medical neglect benefits from the assessment of parental decisional capacity, review of ethical standards and principles.

**Conclusion:** Recognition of the importance of parental decision-making capacity as relates to parental autonomy and medical neglect and understanding key ethical principles will enhance the CAP's capacity in medical decision-making when stakes are high and absolute recommendations are lacking.

## Introduction

With few exceptions, youth under 18 years of age are generally considered to lack capacity and legally cannot provide consent to medical or psychiatric care. Parents/legal guardians are typically viewed as best suited to make treatment decisions for their minor children and as most inclined to act in their child's best interest (1). Child and adolescent psychiatrists (CAPs) treat dependent minors, and it is the parents or guardians who seek services from the CAP. This unique situation creates the potential for ethical conflicts to arise, in that the CAP has obligations to both the minor patient and to the youth's guardian(s). The first principle in the American Academy of Child and Adolescent Psychiatry (AACAP) Code of Ethics is the developmental perspective, which underscores the CAPs duty to “optimize the emotional, cognitive, social and physiological development of all children and adolescents” (2). The developmental perspective is to be incorporated into the CAP's considerations and actions and has implications for the implementation of treatment recommendations. Serious ethical dilemmas arise when the parental right to make decisions about their child (*parental autonomy*) conflicts with the CAP's moral obligation to promote the pediatric patient's welfare (*beneficence*), and provide treatment consistent with best practices. Given the inherent nature of these obligations and likelihood of disagreement regarding treatment recommendations, CAPs are frequently faced with clinical and ethical dilemmas.

Although parental autonomy is widely accepted as the pre-eminent ethical value in the care of minors, the construct of parental decisional capacity provides an important lens in which to view parental autonomy. Decisional capacity is characterized by four key factors: (1) *the ability to demonstrate a consistent preference over time*, (2) *factual understanding* of the situation and treatment proposed, (3) *appreciation of the significance of the information presented*, and (4) *rational manipulation of information* (3). Inability to perform any of the four tasks may result in medical neglect, defined by the American Academy of Pediatrics (AAP) as “… the inability to heed obvious signs of serious illness or follow through a physician's instructions once advice has been sought” (4). Five components have been identified by AAP as necessary to diagnose medical neglect: (1). *A child is harmed or is at risk for harm because of lack of health care;* (2). *The recommended health care offers significant benefit to the child;* (3). *The anticipated benefit of the treatment is significantly greater than its morbidity;* (4). *It can be demonstrated that access to health care is available and not used;* (5). *The caregiver understands the medical advice given* (4).

Medical neglect accounted for 0.8% of reported child maltreatment cases in the United States and accounted for 8.1% of child neglect deaths in 2018 (5). This statistic is likely an underrepresentation, as children who experience medical neglect along with another subtype of maltreatment (i.e., physical or sexual) are reported in a separate, combined category making it difficult to fully appreciate the impact of medical neglect (5). The lack of disease-specific guidelines for managing or reporting medical neglect and wide variation among state reporting requirements have added to the complexity of determining medical neglect (6). Mental health neglect, defined as “*limiting a child's access to necessary mental health care because of reasons other than inadequate resources*” (4) does not capture the complexity of the factors that may contribute to its diagnosis. Consequently, CAPs may experience a lack of support for their advocacy of minor patients given the limitations of the definition and the lack of attention mental health neglect has received in the literature. A review of available literature yielded only two articles. One article addressed parental medical neglect in the treatment of pediatric depression, and the other examined two case reports of parental medical neglect in the treatment of anorexia nervosa (7, 8).

From an ethics perspective, three main ethical standards have been applied to pediatric cases when issues regarding medical neglect and parental autonomy arise. These are the *best interest standard (BIS), harm principle (HP)*, and *constrained parental autonomy (CPA)*. The BIS articulates the primacy of the child's interests, “protecting the moral claims of children against being undermined or reasonably set aside” (9). The HP delineates the condition for state action to override parental decision-making using the threshold of increased likelihood of imminent harm to the child, in contrast to the child's best interest (1). CPA respects the rights of parents to raise their child according to their values unless their decisions do not promote their child's basic needs and interests (10).

## Methods

Two de-identified cases are presented to illustrate the significant clinical and ethical challenges that arise when parental decisional capacity is questioned in light of nonadherence with recommendations for pediatric psychiatric treatment. Each case is analyzed with respect to AAP's criteria for medical neglect and the four pillars of decisional capacity. The ethical standards relevant to medical neglect as well as core medical ethics principles are discussed. Practical guidance is offered for CAPs and treating clinicians facing similar situations.

## Case 1

An adolescent was referred to the outpatient child and adolescent psychiatry clinic for odd affect, cognitive blunting and psychomotor retardation by a pediatric neurologist who ruled out an underlying neurologic condition upon the request of the patient's legal guardian. The legal guardian was a significantly older sibling who had been independently raising the patient over the last several years. As the adolescent was unaccompanied to the psychiatric appointment it was not possible to elucidate the development of the clinical findings over time. Further assessment was delayed due to a missed follow-up appointment and unreturned phone calls by the guardian. Case management was established after a report concerning for medical neglect was filed by the outpatient CAP. With intensified follow-up, concerns mounted regarding the patient's weight and eating habits, given the BMI drastically dropped from the 50th to the 3rd percentile in 6 months, with clinical signs of an eating disorder. Adequate food availability at home was confirmed by the case manager. Behavioral interventions and guidelines for close monitoring were outlined by the CAP; however, these were not adhered to at home despite numerous care conferences between the CAP, legal guardian, case manager, and school counselor. The patient demonstrated lack of insight into the illness and endorsed a lack of motivation for change. Psychiatric symptoms progressed to social withdrawal, and self-imposed seclusion in the context of minimal oral intake. The guardian repeatedly vocalized hopelessness considering the patient's unwillingness to eat and hesitation to follow through with the treatment plan due to concern that the patient may run away from home, although the patient had never expressed this intention. Over the course of treatment, the patient was medically hospitalized on multiple occasions due to malnutrition and unstable vital signs. While weight restoration in the hospital occurred without difficulty, weight drastically plummeted soon after discharge home. Reports of concern for ongoing medical neglect were raised to CPS but were not substantiated by the agency.

### Case 1 Analysis

AAP's criteria 1–4 for medical neglect are met. The patient experienced malnutrition and unstable vital signs (criterion 1). Most would agree that the benefits of behavioral interventions in context of an eating disorder, namely limit setting and consequences for refusal to eat, are significant (criterion 2) and outweigh the risk of the patient's anger over such limit setting (criterion 3). The access to outpatient providers who wish to work with the patient and the guardian, as well as insurance coverage to do so, satisfies criterion 4.

Criterion 5 (*the caregiver understands medical advice given)* warrants further reflection as the guardian's severely compromised ability to implement treatment recommendations was not clearly due to a lack of understanding those recommendations. Factual understanding is only one of the four pillars of decisional capacity and is not enough to ensure the caregiver is able to make decisions for a minor patient. In this case, it was the lack of *ability to appreciate the significance of the information* about the child's clinical situation (the patient's lack of insight regarding the illness) and limited *ability to rationally manipulate information* (i.e., the concern that by setting consequences the guardian would inadvertently push the patient to run away) which resulted in harm to the patient. Boos and Fortin argue that AAP's criterion 5 (along with criterion 4) “*do not truly differentiate between neglected children or not*,” but rather addresses the etiology of the medical neglect. The authors suggest that medical neglect be considered when criteria 1–3 are met (11).

## Case 2

An adolescent was referred to the psychiatric emergency department due to the pediatrician's concerns for psychosis during a routine sports physical. The evaluation revealed irritability, flight of ideas, psychomotor agitation, grandiose delusions, response to internal stimuli, and disorganized speech. Consent for psychiatric hospitalization and medication initiation was obtained from the patient's legal guardian, a single parent. Collateral information obtained from the patient's teacher and school counselor suggested the presence of prodromal symptoms a year prior, with an episode of psychosis without obvious mood symptoms during the previous academic year. School staff highlighted the parent's rationalization of symptoms as a reaction to psychosocial stressors, which was also prominent throughout hospitalization. The treatment team attempted to form a therapeutic alliance with the patient's parent and provide psychoeducation on schizoaffective disorder, its course, prognosis, and treatment. Despite this, the parent rejected the diagnosis and requested early discharge against medical advice. This was honored as the patient's response to internal stimuli, reality testing, and overall function had improved with psychotropic agents (a mood stabilizer and an antipsychotic). The parent began tapering the patient off of the psychotropic agents without medical guidance immediately after discharge, perceiving them toxic and unnecessary.

Several months later, the symptoms recurred and hospitalization was pursued, again upon the recommendation of the patient's pediatrician, with a similar course and outcome. Several days after discharge, the patient was again brought to the emergency department by police due to an uncharacteristic episode of severe agitation at school. Medication nonadherence was inferred, based on subtherapeutic mood stabilizer levels, as a causal factor in this and each of the subsequent three psychiatric admissions, which occurred over a several-month period and with progressively more serious presentations (with delirious mania and catatonia). Throughout treatment the parent discussed matters related to diagnosis and treatment recommendations with clear overestimation of understanding and knowledge of the clinical situation, and frequently challenged the treatment team's recommendations. The parent continued to identify the patient's restlessness as “*nervousness”* around strangers, delusions as “*humor,”* and hypersexuality and intrusiveness as “*friendliness.”* Parental underestimation of the seriousness of psychosis and mania, overestimation of ability to provide adequate supervision to the patient in the outpatient setting without treatment, and lack of appreciation as to the deleterious consequences of untreated or undertreated symptoms on future likelihood of symptom response and remission were the concerns highlighted to Child Protective Services (CPS) by the treatment team; however, medical neglect was not substantiated by the CPS agency.

### Case 2 Analysis

As in case 1, AAP's criteria for medical neglect 2–4 are easily met. However, how one defines harm and lack of healthcare can generate diverging opinions relative to criterion 1 (harm due to lack of healthcare) (9, 11). Unlike the outcome of refusal to consent for chemotherapy or a blood transfusion (or insulin treatment when applied to a more chronic condition), lack of psychiatric treatment does not generally result in death. Notable exceptions, of course, are hospitalization in the context of acute suicidality and electroconvulsive treatment for catatonia. Failure to treat and failure to adequately maintain continuing therapeutic interventions in cases of childhood psychiatric illness, however, can result in harm as evidenced by profound negative long-term sequela, including loss of cognitive capacity and significantly reduced lifespan (12). The whole is greater than the sum of its parts; it is the collection of independently non-life-threatening neglectful decisions that truly pose harm to the population of youth with significant mental health disorders, warranting an expansion of the definition of harm beyond acute, immediate life-threatening situations.

Concern for lack of parental capacity for decision making, raised in this case by parental inability to *demonstrate a consistent preference* of the use of medication (i.e., consenting to psychotropic management in the hospital and discontinuing after discharge); a significant deficit in *factual understanding* (of the diagnosis of schizoaffective disorder and indication of medications used); lack of *appreciation of the significance of information presented* (misattributing psychiatric symptoms to the patient's personality); and subsequently the inability to *rationally manipulate this information*, yet again poses a problem for the fifth criterion. AAP's guidelines place the onus of responsibility on the provider to address any communication barriers or parental medical illiteracy so that the parent may provide informed consent; however, do not offer guidance in the event that such factors are not amendable due to the lack of decisional capacity.

## Discussion

The ethical tensions in both cases illustrate the conflicting ethical principles of parental autonomy to make medical decisions for the minor child and the CAP's professional code of ethics to demonstrate benevolence and obligation to treat the patient. Both cases reflect progressive concerns of the minor patient's well-being and attempts made by the treatment teams to resolve disagreements with parent/legal guardian surrounding their care. The characteristics of chronic, complex and unstable medical conditions present in these cases have been recognized as creating the perfect storm that sets the stage for more chances for medical neglect to occur, and for the outcomes of neglect to be quite detrimental (13). The cumulative effects of parental inability to follow through with treatment recommendations is considered by the treatment team to endanger the long-term physical and emotional well-being of the patient. Furthermore, the inability to follow through with recommendations is considered secondary to a compromised parental/guardian decisional capacity rather than malicious or selfish reasons.

There is debate within pediatric ethics as to the preferred ethical standard to be given precedence in challenging situations with regards to parental autonomy, harm to the child, and questions of medical neglect. How do the main pediatric ethical standards address parental autonomy and parental decisional capacity in the context of chronic illness? Parental decisional capacity is not explicitly discussed in the prevailing ethical standards. We support the application of the best interest standard, as it prioritizes the best interests of the child, and protects the well- being of psychiatrically ill children who often suffer from conditions of longer durations, and who are at risk for or have experienced medical neglect. The BIS can serve as tool for clinicians to help define what is most critical in the treatment of a child (13). The harm principle supports state interference only during imminent harm, excluding the risk for medical neglect associated with chronic illness. To protect parental autonomy, courts grant permission for treatment over parental objections typically in situations where illness or injury is potentially life-threatening (1). Diekema argues, “when a parental refusal does not place a child imminently at significant risk of serious harm, state intervention should be postponed, and attempts made to work with the child's parents or guardians in a non-confrontative manner to resolve the issue” (1). The HP standard, however, does not meet the needs of pediatric patients with severe and persistent psychiatric illness. As criticized by Bester, it “sets the bar too low.” Bester claims “parents owe their children much more than harm avoidance,” “by using only serious imminent harm as a limiter, we would have to accept some seriously inadequate decisions,” and views the best interest standard as the best standard to use in pediatric ethics (9). The constrained parental authority framework states that parents should be able to raise their children in keeping with their own values but are constrained by the basic interests of their children. The term basic interests is open to interpretation, with different value judgments that may not completely align with clinical rationale for course of action and medical decision-making (9).

CAPs appreciate the unique and vital role of parents, the primacy of the parent-child relationship, and often work to strengthen healthy bonds between children and their parents. However, the actions taken by child psychiatrists will be driven by their professionalism, adherence to ethical principles, and sense of duty to act accordingly on behalf of the minor child, especially when failure to act can result in serious harm. CAPs should incorporate the assessment of parental decisional capacity into their practice and re-assessments should occur throughout treatment, particularly as new diagnostic interventions or treatment recommendations are introduced. Children whose parents/legal guardians lack such decisional capacity should be protected against harm as adult patients who lack capacity are protected against harm by the appointment of a surrogate decision- maker. We concur that AAP's first three criteria for medical neglect are the most relevant in diagnosing medical neglect. Rather than using the fifth criterion, we encourage CAPs instead to assess for parental decision-making capacity.

## Conclusion

This paper adds to the limited literature on psychiatric neglect. The use of case illustrations serves to underscore the concern that the harm principle as applied to medically ill children may significantly miss the mark in protecting children with psychiatric illness from serious, albeit longitudinal, harm. Medical neglect as applied to pediatric psychiatric conditions may be significantly underrecognized and underreported, and thus, lead to mistreated, undertreated or untreated psychiatric disorders. The AAP criteria for diagnosing medical neglect creates an unintended consequence with criterion 5, in that if a parent/caregiver does not understand the advice given, the threshold to diagnose medical neglect is not met. Child and adolescent psychiatrists and their pediatric medical colleagues are urged to consider the role of parental decisional capacity assessments and appreciate the strengths and limitations of the three prevailing pediatric ethical standards.

## Data Availability Statement

The original contributions presented in the study are included in the article/supplementary material, further inquiries can be directed to the corresponding author/s.

## Author Contributions

EB, JV, JS, and MR contributed to the formulation of vignettes. EB, GE, MM, and AM contributed to the writing of the manuscript. All authors contributed to the article and approved the submitted version.

## Conflict of Interest

The authors declare that the research was conducted in the absence of any commercial or financial relationships that could be construed as a potential conflict of interest.
